# Renin-angiotensin inhibitors reprogram tumor immune microenvironment: A comprehensive view of the influences on anti-tumor immunity

**DOI:** 10.18632/oncotarget.26174

**Published:** 2018-10-26

**Authors:** Dora L. Vallejo-Ardila, Theodora Fifis, Louise M. Burrell, Katrina Walsh, Christopher Christophi

**Affiliations:** ^1^ Department of Surgery, Austin Health, University of Melbourne, Melbourne,VIC 3084, Australia; ^2^ Department of Medicine, Austin Health, University of Melbourne, Melbourne, VIC 3084, Australia; ^3^ Department of Cardiology, Austin Health, University of Melbourne, Melbourne, VIC 3084, Australia

**Keywords:** renin-angiotensin system, tumor microenvironment, anti-tumor immunity, kallikrein kinin system

## Abstract

Renin-angiotensin system inhibitors (RASi) have shown potential anti-tumor effects that may have a significant impact in cancer therapy. The components of the renin-angiotensin system (RAS) including both, conventional and alternative axis, appear to have contradictory effects on tumor biology. The mechanisms by which RASi impair tumor growth extend beyond their function of modulating tumor vasculature. The major focus of this review is to analyze other mechanisms by which RASi reprogram the tumor immune microenvironment. These involve impairing hypoxia and acidosis within the tumor stroma, regulating inflammatory signaling pathways and oxidative stress, modulating the function of the non-cellular components and immune cells, and regulating the cross-talk between kalli krein kinin system and RAS.

## INTRODUCTION

**Figure 1:**
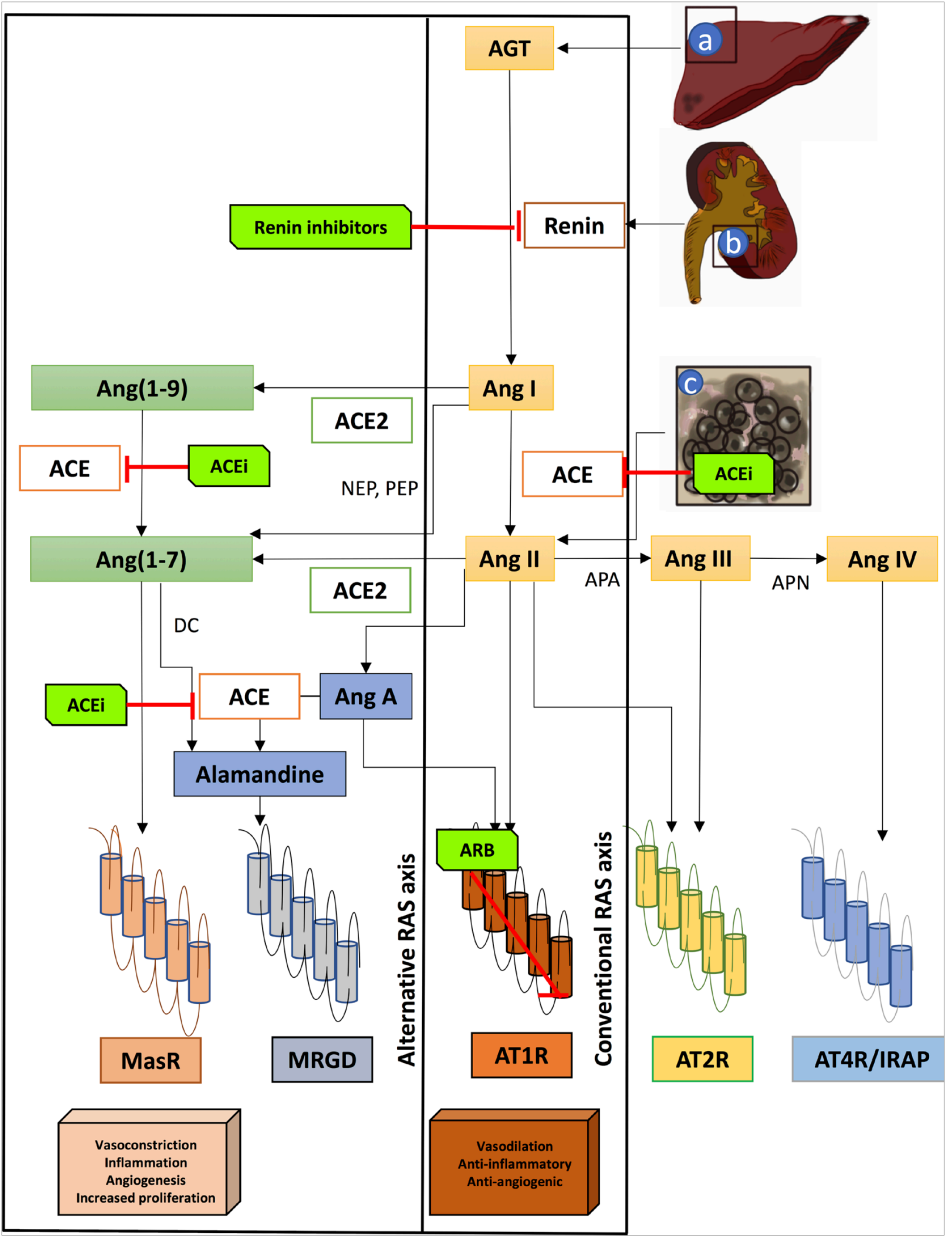
The pro-tumor and anti-tumor mediated effects of the conventional and alternative RAS axis Angiotensinogen (AGT) is produced and released into circulation by the liver **(a)**, then AGT is hydrolyzed by renin. Renin is produced by the juxtaglomerular cells of the kidney **(b)** to form Angiotensin I (Ang I). Afterwards Ang I is hydrolyzed by angiotensin-converting enzyme (ACE) in the endothelial cells of the lungs **(c)**, to produce the biologically active angiotensin II (Ang II). Ang II interacts with two different receptors, angiotensin II type 1 receptor (AT1R) and angiotensin II type 2 receptor (AT2R). ACE2 cleaves Ang II to Angiotensin (1-7) (Ang (1-7)), whereas ACE produces Ang (1-7) by cleaving Angiotensin (1-9) (Ang (1-9)). Ang (1-7) commonly acts via mitochondrial assembly receptor (Mas receptor or MasR). Alamandine is generated either by the cleavage of Ang A or Ang (1-7), by ACE and decarboxylase (DC) respectively. Alamandine binds through MAS-related G protein couple receptor D (MRGD). Angiotensin II is metabolized to Angiotensin III (Ang III) by aminopeptidase A (APA), whereas Ang III is metabolized to Angiotensin IV (Ang IV) by aminopeptidase N (APN). AT4R or Insulin-regulated membrane aminopeptidase (IRAP) is activated by Ang IV. NEP, neutral endopeptidase; PEP, prolyendopeptidase. The inhibition of the conventional axis by ACEi or ARB reduces inflammation, vasoconstriction, and angiogenesis means by which also inhibits tumor growth. Opposite effects have been associated with the activation of the alternative axis including vasodilatation, antinflammatory and anti-angiogenic effects.

The main components of the conventional axis of the renin-angiotensin system (RAS) [such as angiotensinogen (AGT), renin, angiotensin-converting enzyme (ACE), angiotensin I (Ang I), angiotensin II (Ang II)], function as an intricate peptide signaling network through several receptors [including angiotensin II type 1 receptor (AT1R) and angiotensin II type 2 receptor (AT2R)] [1]. AGT is produced and released into circulation by the liver, then AGT is hydrolyzed by renin, which is produced by the juxtaglomerular cells of the kidney to form Ang I [1]. Afterwards Ang I is hydrolyzed by ACE in the endothelial cells of the lungs, to produce the biologically active Ang II. Ang II interacts with two different receptors, AT1R and AT2R [1]. The alternative axis comprises ACE2, which is known as ACE-related carboxypeptidase or angiotensin-converting enzyme homolog (ACEH). ACE2 is mostly found in the vascular endothelial cells and renal tubular epithelium [2] ACE2 cleaves Ang II to Ang-(1-7), whereas ACE produces Ang-(1-7) by cleaving Ang-(1-9). Ang-(1-7) commonly acts via mitochondrial assembly receptor (Mas receptor or MasR) [3]. Another peptide alamandine is generated either by the cleavage of Ang A or Ang-(1-7), by ACE and decarboxylase (DC) respectively. Alamandine binds through MAS-related G protein couple receptor D (MRGD) [3]. Several other truncated bioactive peptides have been previously characterized including Angiotensin III [4], Angiotensin IV [5], and Ang A [6], as part of the RAS network (Figure 1).

In the mid-1970s it became possible for the first time to therapeutically target the components of Ang II/AT1R axis using the angiotensin-converting enzyme inhibitor, captopril [7], and followed by selective blockade of the AT1R with losartan [8]. Renin-angiotensin system inhibitors (RASi) includes two classes of pharmacological agents, angiotensin-converting enzyme inhibitors (ACEi) and angiotensin II receptor blockers (ARBs) [8]. A third class of RASi, a direct renin inhibitor such as aliskiren is also included in the list of RASi approved by the U.S. Food and Drug administration (FDA) [9].

RASi are widely used to treat heart failure [10, 11], myocardial infarction [12], and systemic hypertension [13] and have proved to be effective in preventing cardiovascular [14] and renal associated comorbidities [13]. Additionally RASi have shown potential anti-tumor effects that promise a significant impact in cancer therapy [15]. Several studies have yielded diverging findings on the role of RASi on the incidence of recurrence, metastasis and survival in cancer patients [16]. To comprehend the potential benefit of RASi in cancer patients consideration should be given to factors such as tumor heterogeneity, tumor stage, hormone receptor status, human epidermal growth factor receptor 2 (EGFR2) over-expression, and (neo)adjuvant treatment regimen [15]. A meta-analysis including 55 studies suggested that RASi may improve the survival of cancer patients depending on cancer type and class of RASi [16].

Differences between the mediated effects of the RAS conventional and alternative axis has been previously described. In general, the over-expression of RAS components within the Ang II/AT1R axis (such as ACE and AT1R) is associated with tumor growth and with more aggressive tumor features in several types of human cancer, including breast cancer, ovarian cancer and renal cancer [17–19], whereas Ang II/AT2R and Ang(1-7)/MasR showed opposite effects [20]. Nonetheless there are also conflicting evidence, which may suggest tumor-type specific differences [18]. Similarly, ACE2/Ang-(1-7)/MasR axis dysregulation has been shown to be up-regulated or down-regulated depending on the type of cancer [21–23] (Figure 1).

The role of the various RASi in modulating pathological processes involving cell inflammation [24, 25], fibrosis [26–28], and tumor growth [29–32], has been well-documented, but its influence on anti-tumor immunity is uncertain. The aim of this literature review is to analyze current evidence on the effects of RASi on anti-tumor immunity by reprogramming the tumor microenvironment (TME).

**Figure 2:**
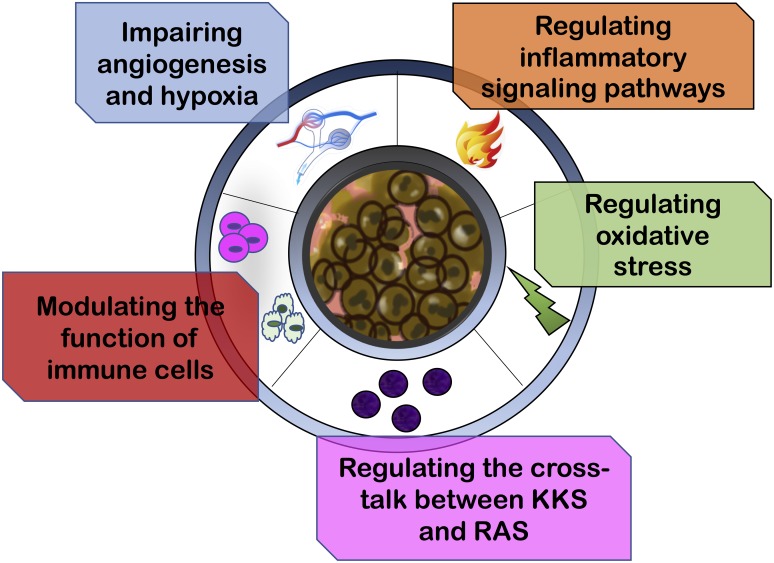
Renin-angiotensin inhibitors reprogram tumor immune microenvironment The pathological processes and cellular functions inside the tumor microenvironment which appear to be influenced by RASi include tumor angiogenesis, hypoxia and acidosis within the tumor stroma, inflammatory signaling pathways, oxidative stress, immune cell modulation and the role of kalli krein kinin system (KKS).

The pathological processes and cellular functions inside the TME which appear to be influenced by RASi include tumor angiogenesis, hypoxia and acidosis within the tumor stroma, inflammatory signaling pathways, oxidative stress, immune cell modulation and the role of kalli krein kinin system (KKS) (Figure 2).

### Regulation of tumor angiogenesis using renin-angiotensin inhibitors impacts cancer progression

Conclusive evidence has shown that targeting Ang II/AT1R impairs neovascularization and vascular permeability and decreases microvessel density by reducing vascular endothelial growth factor (VEGF)-expression [19, 33–35]. Angiogenesis is initiated by the disruption of the endothelial cells (EC) monolayer, and their invasion into the surrounding stroma. The recruitment of pericytes along the basement membrane into the new vessels provides both, mechanical stability and molecular cross talk with EC, which induces VEGF production and survival signaling [36, 37]. The cellular infiltrating populations into the TME can originate either in the blood vessels, the stroma or the bone marrow [37]. For example, cancer-associated fibroblast (CAFs) can be derived from both, tumor stroma and from bone marrow precursors [37]. This cellular plasticity is mediated by the epithelial-mesenchymal transition (EMT) [37]. Many of the different cell types involved in tumor vasculature, and-/or are part of the TME such as pericytes [38, 39] and CAFs [40] express RAS components. Regulating angiogenesis using RASi may extend beyond the converging point of VEGF-response. ACE2 acts as potent counter-regulator against ACE and Ang II activity [20, 41, 42]. ACE2 overexpression or ACE2-Ang-(1-7)- MasR activation may suppress angiogenesis either by inhibiting the production of VEGFa in NSCLC [43], or VEGF receptors attenuation in nasopharyngeal carcinoma [44], respectively.

The use of RASi concomitant with anti-VEGF therapy was associated with better survival in metastatic renal cell carcinoma [45–48], metastatic CRC [49], advanced HCC [50] and glioblastoma [51]. Increased resistance has been reported to VEGF inhibition [52, 53]. The tumor vasculature interacts with the TME via several other mechanisms offering potential new therapeutic targets [54]. The regulation of TME by emerging anti-angiogenic therapies are aiming to counteract tumor progression and to achieve greater tumor destruction. Rather than acute vessel disrupting strategies, there is now a focus on anti-inflammatory regimens. For instance, cabozantinib inhibits the activity of VEGFR, c-MET, AXL and other tyrosine kinases, thereby leading not only to impair angiogenesis but also the disruption of other processes inside the TME [55].

### Targeting Ang II/AT1R axis to impair hypoxia and acidosis within the tumor stroma

Local RAS works synergistically and independently of systemic RAS in a paracrine fashion [56]. Ang II mediates effects that reduce tumor perfusion and oxygenation, resulting in hypoxia and subsequent acidosis within the tumor stroma [57]. For example, local Ang II, predominantly exists in hypoxic regions of nasopharyngeal carcinoma and breast cancer cells, where it is autocrinely produced by chymase-dependant rather than ACE dependent mechanism [58]. In which case the action of ACEi will not be effective to inhibit tumor growth. Tumor hypoxia and acidosis trigger a cascade of up-regulation of transcription factors, growth factors and cytokines, including hypoxia inducible factor (HIF), VEGF and Transforming growth factor beta (TGF-β) that together promote immunosuppression within the TME [59, 60]. An immunosuppressive microenvironment includes impaired function of T cells [61], dendritic cells [62] the accumulation of M2-like macrophages [63] and MDSC, but also increased expression of immune check point molecules such as PD-L1 / PD-1 on tumour / stromal cells and the immune cells [64].

In the liver, the local RAS is up-regulated in response to tissue injury and hypoxia [31]. The main effector is Ang II, which normally maintains tissues homeostasis, in this scenario can stimulate the expression pro-angiogenic VEGF via AT1R signaling, leading to wound healing in tissue injury or to tumor neovascularization in hypoxic tumors [65]. ACEi mechanism of action is to reduce the production of Ang II, while ARBs selectively block the action of AT1R receptors inhibiting tumor-associated angiogenesis [16].

### Renin-angiotensin inhibitors regulate inflammatory signaling pathways and oxidative stress in tumor biology

The TME is bound to the dynamic between malignant and non-transformed cells [66]. Non-malignant cells are reprogrammed to accomplish tumor-promoting functions during all stages of carcinogenesis [66]. Intercellular interactions modulate the chemical and physical properties of any tissue through a diverse pool of secreted cytokines, chemokines, growth factors, and inflammatory or matrix remodeling enzymes [67]. TGF-β suppresses the differentiation and function of T helper ( T_H_), CD8^+^ cells, Natural Killer (NK) cells, and tumor-associated neutrophils (TANs), tumor associated macrophages (TAMs) and myeloid-derived suppressor cells (MDSCs) [67]. Tumor supporting cytokines are released from tumor and stromal cells upon AT1R activation via Ang II including, TGF-β Interleukins (IL-1a, IL-1B, IL-6, IL-8) and MCP-1: (monocyte chemoattractant protein 1), macrophage colony-stimulating factor (M-CSF), cyclooxygenase 2(COX-2), C- reactive protein (CRP) [68]. Immunomodulatory cytokines may up-regulate immunosuppressive pathways, i.e. COX-2 via prostaglandin E2 synthesis, and impair dendritic cell (DC) function by reducing their migration [68, 69]. Ang II/AT1R signaling induces reactive oxygen species (ROS) generation and related proteins such as inducible nitric oxide synthase in the tumor cells and stroma cell [70]. Exposure to ROS in the TME can impair T cell function while enhancing T regs and TAMs, as has been previously reported in prostate cancer [69]. Treatment with the ARB candesartan diminishes ROS generation [71].

### Renin-angiotensin inhibitors modulate the function of immune cells within the tumor microenvironment

There are three major immunosuppressive cell types within the tumor mass; MDSCs, TAMs and CAFs. MDSC include both myeloid progenitors cells and immature myeloid cells [72]. These cell have been characterized as a population of inhibitory immune cells that lack typical mature myeloid markers, in mouse and human cancers [72]. Murine and human MDSCs exert their suppressive effects through several mechanisms that inhibit CD8+T cell activation [73], Additionally they induce the development of T regs population, the polarization of macrophages to a TAM-like phenotype, the production of ROS, the upregulation of nitric oxide and depletion of nutrients via increased activity of L-arginase [73]. ACE is required for normal myelopoiesis, as an increased level of CD11(+) bGrl (+) cell-similar to MDSCs phenotype were detected after extramedullary myelopoiesis compensatory to bone marrow incompetence [74]. Likewise, ACE may have a role in the accumulation of MDSCs in the TME. However, the inhibitory function of MDSCs over CD8+T cells in the TME requires further investigation [74].

Similarly, TAMs are usually pro-tumorigenic and have shown that a polarized M2-like phenotype contributes to immunosuppression, whereas M1-like phenotype induces anti-tumor immunity [63]. TAMs tend to accumulate in hypoxic and/or necrotic areas of the tumor [75]. Studies with ACE KO and ACE 10/10 mouse model indicated that ACE plays key role in macrophages to regulate the production of pro-inflammatory cytokines such as IL-12, nitride, and TNF-α in response to lipopolysaccharide (LPS) or chitin (a polymer of *N*-acetylglucosamine). These induce M1 macrophages or activate M2, respectively [76]. Additionally, M1 induction through granulocyte-macrophage colony stimulating factor (GM-CSF) upregulate ACE expression in human monocytes [76, 77]. Recent findings remain inconclusive as to how RAS modulation establishes M2 macrophage induction and polarization. Some studies suggest that there is no difference between ACE KO, ACE 10/10 and WT model [74]. Others have found an increased or decreased M2 response by RAS blockade [78, 79]. Our laboratory has shown the immunomodulatory role of RAS in influencing TAMs. Blockade of ACE with captopril increased Kupffer cells infiltration in the tumor-bearing liver during an early stage of tumor progression in CRC liver metastasis [80].

Myofibroblasts also known as CAFs are derived from multiple precursors, such as myoepithelial cells, mesenchymal stem cells, smooth muscle cells and EC [81]. Their capability to secrete EGF family growth factors hepatocyte and fibroblast growth factors and insulin growth factor confer them the potential to induce malignant transformation of cells [82, 83]. CAF express AT1R [40], through which Ang II [40] stimulates their proliferation and induces the production of various cytokines including TGF-β [40]. TGF-β in addition to its immunosuppressive role as outlined above, can stimulate EMT in malignant cells contributing to an immunosuppressive TME and therapy resistance [84, 85]. Our laboratory found that RAS is implicated in the regulation of EMT in CRC via both AT1 and AT2 receptors by Ang II modulated migration [86]. Additionally, CAF tumor promoting effects have been associated with promoting neovascularization and recruitment of immune cells in the TME via NF-κβ signaling pathway [87]. A recent study concluded that local RAS activity can modulate the function MDSCs, TAMs and CAFs, and indeed is a potent inducer of immunosuppression in the TME [40]. Inhibition of local RAS augments the induction and infiltration of tumor antigen-specific T cells, reduces the T cell suppressive activity of tumor-infiltrating CD11b^+^ cells including MDSCs and enhances the T-cell stimulatory activity of CAFs [47].

In addition to CAFs, other types of immune cells also express RAS components. These include monocytes, neutrophils, dendritic cell and T cells, thus presenting pivotal targets for immune therapy [15]. For example, the stimulatory and coinhibitory interactions between lymphocytes infiltrating tumor stroma and tumor cells expressing receptor-ligands pairs, like immune check point inhibitors [cytotoxic T-lymphocyte antigen-4 (CTLA-4), programmed cell death-1 (PD-1), and its ligand (PD-L1)] correlates with T cell dysfunction [37]. The inhibition of AT1R enhances the induction of tumor antigen-specific CD8^+^ cytotoxic T lymphocytes via gp70-Tcells [40]. Additionally, a clinical study of non-metastatic pancreatic ductal adenocarcinoma (PDAC) patients reported significant overall survival in the chronic RASi users, while unbiased gene profiling of resected tumors from this group presented enriched gene signatures associated with antigen processing and presentation, and activity of T cells, and reduced gene signatures associated with tumour aggressiveness. These findings may suggest that RAS signaling could modulate the efficacy of immunotherapy on this type of cancer [88]. Altogether the synergistic use of RASi combined with immune check point inhibitors may improve anti-tumor efficacy by reprograming the TME towards an immune stimulatory milieu.

### Targeting tumor non-cellular components using renin-angiotensin inhibitors may enhance antitumor immunity

RAS has been studied under the scope of chronic wound healing and fibrosis [89]. During stromagenesis activated fibroblasts, have a critical function in orchestrating ECM remodeling. The effects of tissue renin angiotensin (tRAS) on chronic wound healing can be divided into two opposite axes: (a) proinflammatory/pro-fibrotic and (b) anti-inflammatory/anti-fibrotic [89]. RAS signaling modulates ECM remodeling via AT1 by stimulation in dermal fibroblast, which promotes deposition of fibrotic ECM [90]. The contrary effect was observed via AT2 stimulation, which reduced collagen deposition counteracting AT1 signaling through engagement of tyrosine phosphatase SHP-1 [91]. In the case of tumor-associated stromagenesis, the pathophysiologic response fails to resolve. RAS activated CAFs secrete collagen and other ECM components which leads to a fibrotic reaction so-called tumor desmoplasia [92]. A tumor desmoplastic environment can be either a physical barrier to immune cell infiltration [93], or can provide the substratum to their interstitial migration [94]. Additionally, RAS activated CAFs and other stromal cells produce immunosuppressive cytokines/ growth factors especially TGF-β in the TME leading to chronic inflammation and effector T- cell disfunction, apoptosis and failure to infiltrate deep into the tumour [61]. RASi targeting of the tumor stroma resulted in collagen I synthesis inhibition by CAFs in a dose-dependent manner in different desmoplastic models including human breast, pancreatic, and skin tumors in mice [95]. A collateral effect of decreasing tumor desmoplasia is the decompression of tumor blood vessels and subsequent reduction of tumor hypoxia by increasing perfusion, which may also enhance antitumor immunity [96].

### The cross-regulation of kalli-krein kinin system and renin-angiotensin system by angiotensin-converting enzyme has pleotropic effects in the tumor microenvironment

**Figure 3:**
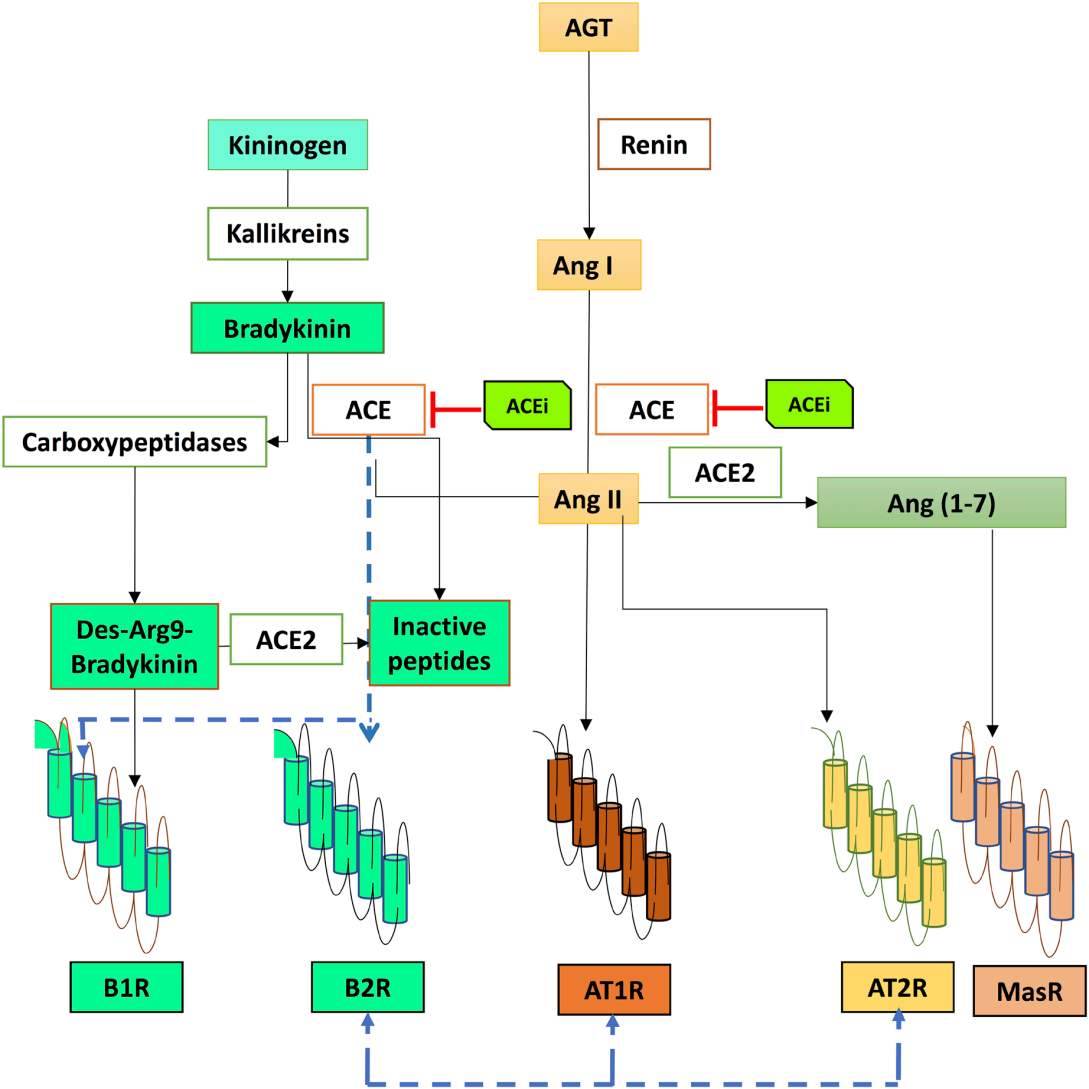
Renin-angiotensin system components intersecting with the kalli krein kinin system Angiotensin-converting enzyme (ACE) is a major hub intersecting between the crosstalk of both systems, by regulating the levels of Angiotensin II (Ang II) and kinins. ACE metabolizes bradykinin (BK), and converts Angiotensin I (Ang I) into Ang II. Interactions between ACE and kinin receptor 1 (B1R) and/or kinin receptor 2 (B2R) have been reported, same as interactions between angiotensin II type 1 receptor (AT1R) and angiotensin II type 2 receptor (AT2R) with B2R.

The KKS comprises several peptides that are produced and cleaved in various sites to finally release the vasoactive kinins [97]. Kinins are originated from their parental molecules, the kininogens [97]. Diverse enzymes known also as kininases such as ACE, neutral endopeptidase (NEP), carboxypeptidase N, carboxypeptidase M, cathepsin X and aminopeptidase metabolize the kinins, [98] (Figure 3). Bradykinin (BK) is the final bioactive peptide produced upon the activation of the KKS, which has pleiotropic functions depending on the pattern signaling triggered through its two different receptors [99]. The kinin receptor 1 (B1R) and the kinin receptor 2 (B2R) activate the same signaling pathway, while inducing a different duration and intensity of the signal modulated by calcium influx [100]. B1R and B2R can be expressed in the same cell type, such as EC, fibroblast and several tumor cells [101]. While B1R expression is induced upon inflammatory conditions and tissue injury, B2R is constituvely expressed [102]. The overall effect of BK through either B1R, B2R, or both is to promote vascular cell proliferation [103], influence barrier permeability [104] and possibly stimulate the release of pro-inflammatory cytokines [105]. The role of kinin receptors has been investigated in various types of cancer, including melanoma, renal, prostate, lung and breast and mesothelioma [106–110], among others. Recently our laboratory found that both human and mouse CRC cell lines showed a strong positive expression of B1R and B2R, and while the inhibition of both receptors delayed tumor growth, only B1R blockade reduced tumor load and increased tumor apoptosis [105]. Indistinctively, blockade of B1R or B2R diminished tumor vascularization *in vivo*, and impaired proliferation and migration of CRC cell *in vitro* [111]. The effect of RASi -ACEi- leads to accumulation of kinins and subsequent B1R activation [112], which may influence not only the cellular components part of the TME by activating macrophages and DC, but also can up-regulate cytokine production of IL-6 and IFN-γ [97]. ACEi activate B2R on DC, with increased production IL-12 [113]. Further studies are required to elucidate the therapeutic opportunities of modulating kinin receptors with ACEi and their influence on the TME.

### Perspectives and significance

Over the last decade a large number of clinical studies have shown the benefits of using RASi in patients at different stages in several types of cancer. The evidence of RASi impairing tumor growth beyond the function of modulating tumor vasculature is rapidly increasing. A major challenge in the field of cancer therapeutics is the increasing rate of resistance to chemotherapy [114], and immunotherapy [115]. A better understanding of the complex interaction between non-cellular components, tumor cells, tissue resident immune cells and infiltrating immune cells within TME is required to develop new cancer treatment strategies. An immunosuppressive TME affects the efficacy of immune checkpoint therapy, which is reflected in two different clinical scenarios, overall survival and adverse side effects. RASi are able to reprogram the TME, using mechanisms by which they impair hypoxia and acidosis within the tumor stroma, regulate inflammatory signaling pathways and oxidative stress, modulate the function of the non-cellular components and immune cells, and regulate the cross-talk between KKS and RAS. Targeting RAS conventional axis (Ang II/AT1R), KKS and enhancing RAS alternative axis (ACE2/Ang-(1-7)/MasR) seem to be promising strategies to effectively influence TME toward an immunostimulatory milieu, and subsequently improve immunotherapy outcome for a larger population of cancer patients. Further pre-clinical and clinical studies are necessary to propose that the concomitant use of RASi and immunotherapy could not only improve the overall survival of cancer patients, but also could decrease the immunotherapy related side effects. Lastly, additional studies may be required in order to conclude if the effects of RASi on the tumor stroma, not only would depend upon the degree of tumor desmoplasia, but also may depend on how they can influence tumor immune infiltration by either impairing or promoting their migration throughout the interstitial space.
